# BOSTON AGING TOGETHER STUDY: ATTITUDES TOWARD OWN AGING AMONG OLDEST-OLD PARENTS AND CHILDREN

**DOI:** 10.1093/geroni/igz038.1704

**Published:** 2019-11-08

**Authors:** Yijung K Kim, Kathrin Boerner, Kyungmin Kim, Daniela Jopp

**Affiliations:** 1 University of Massachusetts Boston, Boston, Massachusetts, United States; 2 University of Lausanne, Lausanne, Switzerland, Switzerland

## Abstract

One consequence of modern longevity is the growing number of older adults with very old parents. While family members are often interdependent in their development and aging, less is known about how intergenerational relationships may influence individuals’ attitudes toward their own aging in later life. Using 70 dyads of oldest-old parents (Mage = 93) and their children (Mage = 67) from the Boston Aging Together Study, we examined the dyadic concordance in positive attitudes toward own aging, and how perceptions of giving and receiving care are associated with attitudes toward own aging for parents and children. On average, parents reported more negative attitudes toward own aging than did children. In less than half of all dyads (46%), both parents and children reported positive (i.e., score three or higher on a scale that ranged from one to five) attitudes toward own aging. T-test results showed that the dyads with positive attitudes toward own aging had more within-dyad age difference, better average self-rated health, fewer depressive symptoms and less loneliness than others. For children, higher level of caregiver’s burden was associated with more negative attitudes toward own aging. For parents, perception of received support was not associated with their attitudes toward own aging. This study sheds light on how both individual and family characteristics may influence individuals’ aging perceptions. Findings suggest the context of parent-child ties may particularly be relevant to those older adults who may have to deal with their own aging- related challenges as well as those of their parents.

